# Prehabilitation of dysphagia in the therapy of head and neck cancer- a systematic review of the literature and evidence evaluation

**DOI:** 10.3389/fonc.2023.1273430

**Published:** 2023-12-18

**Authors:** Sarah Vester, Anna Muhr, Johannes Meier, Christoph Süß, Peter Kummer, Julian Künzel

**Affiliations:** ^1^ Department of Otorhinolaryngology, Department of Phoniatrics and Pediatric Audiology, Hospital of the University of Regensburg, Regensburg, Germany; ^2^ Department of Oral and Maxillofacial Surgery, Regensburg Hospital of the University of Regensburg, Regensburg, Germany; ^3^ Department of Radiotherapy, Hospital of the University of Regensburg, Regensburg, Germany; ^4^ Department of Otorhinolaryngology, Hospital of the University of Regensburg, Regensburg, Germany

**Keywords:** prehabilitation, dysphagia, aspiration, speech therapy, head and neck squamous cell carcinoma, flexible endoscopic evaluation of swallowing

## Abstract

**Background:**

Prehabilitation is becoming increasingly important in oncology because of the significant survival benefits that the reduction of malnutrition provide. Specifically, tumor- and therapy-related dysphagia leads to malnutrition in more than half of head and neck tumor patients. Studies describe the positive effects of an early onset of swallow-specific prehabilitation on the protection of the swallowing function. This paper intents to evaluate the existing evidence on the efficacy of preventive forms of swallowing therapy.

**Methods:**

A systematic literature search was performed in February 2022 in the Cochrane Library, MEDLINE via PubMed, and ClinicalTrials.gov databases for randomized controlled trials investigating preventive swallowing therapy in head and neck tumor patients. This Procedure complies with the PRISMA statement. The RCTs were evaluated by using the PEDro Scale and the Cochrane Risk of Bias tool RoB2.

**Results:**

Five randomized-controlled trials with 423 participants were identified. Four Studies showed moderate to high quality in the PEDro analysis, one showed less. The risk of bias was high in all studies because there was no possibility for blinding and there were high dropout rates. Heterogeneity in interventions, measurement instruments, measurement time points, and outcomes limits a general statement about which swallowing exercises are suitable for the prevention of dysphagia in head and neck tumor patients. Evidence is provided for short-term effects (≤24 months) on functional aspects of swallowing and quality of life. Overall, a decreasing adherence over time was observed in the intervention groups.

**Discussion:**

Initial studies describe swallowing-specific prehabilitation programs in head and neck tumor patients as effective, at least in the short term, whereas long-term effects need to be further investigated. At the current time the evidence base for clear recommendations does not appear to be sufficiently high and studies share a high risk of bias. Further well-designed research, especially considering the conditions in the national health care system, is needed.

**Other:**

There was no funding and no registration.

## 1 Introduction

Latest studies show positive effects of intense pretherapeutic preparations on the outcome of frail and malnourished oncological patients (1, 2). More than a third of hospitalized patients show signs of malnutrition; far more than assumed until now (3). The aim of prehabilitation is to recognize frailty, anemia as well as malnutrition and to improve until the actual therapy starts (4).

The German guidelines for oral-cavity- and larynx-carcinoma do not clearly recommend the structured, therapeutic preparation to secure patients’ nutrition (5, 6), although higher age and multimorbidity of HNC-patients lead to an increased risk of morbidity (7). Therein, the oncology’s focus lies on enhanced therapeutical measures, e.g. intensity-modulated radiation, minimally invasive or reconstructive surgery, deescalating strategies of therapy and the traditional rehabilitation. In Germany the occurring of dysphagia is the starting point of a professional swallowing therapy, mostly in a rehabilitation after the surgery or the chemoradiation (8).

HNC patients particularly have a higher risk for malnutrition as the localization of cancer in the upper pharyngolaryngeal system causes dysphagia. A second risk factor is the unhealthy lifestyle. Other complications of dysphagia are aspiration pneumonia with increasing mortality, social isolation and loss of quality of life (9–11). The prevalence of dysphagia depends on the carcinoma’s localization and size and is up to 80% in HNC patients (12, 13).

Foreign studies present better outcomes for HNC patients if the therapy of dysphagia is started before or during the radiation treatment (10, 13). The idea is that preventive swallowing exercises can reduce the complications of dysphagia that is preexisting or is a consequence of cancer treatment (14).

The aim of this study was to explore if there is evidence of preventive swallowing exercises to maintain swallowing function before and during the primary tumor therapy of HNC patients. Special interest was to see which outcomes and which exercises were useful.

## 2 Methods

An explorative systematic review of the literature was performed. The second author (A.M.) did the literature research in February 2022. This procedure complies with the PRISMA statement (checklist is available in **Supplementary 1**) (15).

According to the criteria of subject focus, document type, possible search and filter functions, and free access to the subject database, the appropriate selection of the databases Cochrane Library, MEDLINE via PubMed, and ClinicalTrials.gov was made. The search language for these databases is English.

The search terms in **Table 1** were chosen by the PICO method, according to the PICO question: How does preventive swallowing therapy (=I) work to conserve the swallowing function (=O) in head neck cancer patients (=P) compared to head neck cancer patients without preventive swallowing therapy (=C), supplemented with timing before tumor therapy (=T) and study type(=S) randomized controlled trials (RCT). The synonymous keywords are linked with the Boolean operator OR, the search components with AND (16).

**Table 1:** Search terms.

Patient P=Head and Neck Cancer	Intervention I=swallowing therapy	Outcome O=swallowing function	Timing T=before cancer therapy	Study type S=RCT
• neck and head cancer • Cancer of head and neck • cancer of the head and neck • cancer of neck and head • cancer of the neck and head • cancer of neck • cancer of the neck • head and neck neoplasm • head and neck neopl* • neck cancer • neck neoplasms • neck neoplasm • squamous Cell Carcinoma of Head and Neck	• speech and language • disease management • treatment	• deglutition • dysphagia	• preventive • prophylactic • during • before • preoperative • prehabilitate*	• randomized controlled trials (RCT)

Multiple trial searches of the MEDLINE database via PubMed were performed to verify and appropriately adjust the search strategy before the search. The database indicated errors such as incorrect bracketing or use of the stop words “and, during, before, and the”. Accordingly, the search syntax was edited. In addition to correcting typos and bracketing, major revisions included adding the search component (swallowing OR deglutition OR dysphagia) in conjunction with the AND operator to exclude studies in which dysphagia did not represent study content. The Peer Review of Electronic Search Strategies (PRESS) checklist (17) was used for final review of the search string. Depending on the database the search matrix was adapted (as seen in **Supplementary Material 2**).

Using the inclusion and exclusion criteria shown in **Table 2**, library records were selected, and duplicates were sorted out. Publications that did not answer the research question were excluded from the further search. These included studies that examined medication, administration or different doses of radiation therapy as an intervention instead of exercise therapy measures, as well as studies that did not assess swallowing function as an outcome. The inclusion criterion that participants were HNC patients had to be met, so studies in patients with esophageal cancer were excluded. Furthermore, results were excluded if they were not randomized controlled trials. Also excluded were studies registered on ClinicalTrials.gov whose outcome data could not be viewed.

**Table 2:** Inclusion and exclusion criteria of the systematic literature research.

Inclusion Criteria	Exclusion Criteria
• Randomized controlled trial (RCT) • Intervention: physical exercise to improve or preserve the swallowing function before or during the primary therapy (operation or radiotherapy/chemoradiation) • Primary diagnosis of HNC • Control group without swallowing exercises • Full-text available in English	• Swallowing function not evaluated • RCTs without results or no access to read the results • Intervention: no swallowing exercises

For reasons of transparency and to secure the search, the hits were exported to the literature management program Citavi 6 (Swiss Academic Software GmbH; Wädenswil, Swiss). The assignment into categories allows a selection.

After identifying eligible studies, the most important data and results were extracted and summarized. Particular emphasis was placed on the type of intervention in the comparison groups, as well as the outcome parameters and timing of outcome measurement. Statistically significant results were highlighted. (see **Tables 3**, **4**).

**Table 3:** Overview of interventions and outcome measures of the RCTs (18–22).

Study Country Number n	Age (MW) Tumor-localization Tumor stage Therapy	Intervention	Outcome after 6 weeks	Outcome short term after 2-3 month	Outcome mid term after 4-6 month	Outcome long term after 7-12 month
**Hajdú et al.** (18) **DK** **n = 235**	38-88 (63) Pharynx, Larynx, Oral cavity, CUP UICC I-IVb Curatively intended Radiotherapy	I: 2x/week physiotherapy, 3x/week swallowing therapy with occupational therapist, 3x/day self-administered swallowing exercises during radiation C1: Usual treatment, occuptional therapist. (1x/week) (active group) C2: No treatment (non-active group)	• **^I^mouth opening** • wight • FOIS • MDADI • depression • **^I^anxiety(SCL-92)** • **^I^pain** • **^I^EORTC-QLQ-C30** • **^I^EORTC-HN-35**	• mouth opening • wight • PAS • **^c^Yale Scale** • FOIS • MDADI • depression • anxiety (SCL-92) • pain • **^I^EORTC-QLQ-C30** • **^I^EORTC-HN-35**	• mouth opening • wight • FOIS • MDADI • depression anxiety (SCL-92) • pain • EORTC-QLQ-C30 • EORTC-HN-C35	• mouth opening • wight • PAS • Yale-Scale • FOIS • MDADI • depression • anxiety (SCL-92) • pain • EORTC-QLQ-C30 • EORTC-HN-C35
**Messing et al. (** 21) **USA** **n = 60**	39-79 (56) Oral cavity, Pharynx, Larynx UICC III -IV Chemoradiotherapy	I: 2x/day Swallowing exercises; oromotor strength/strength exercises and swallow maneuvers, during CRT and 3 month post CRT, 1x/week swallow therapy c: No swallow therapy, TheraBite (Usual care)	not evaluated	• mouth opening • wight • oromotor assessment • CTCAE mucositis and oral ulceration • Gastric feeding tube • **^I^OPSE** • **^I^pharyngeal phase impairments** • PAS • FOIS • pain • **^I^EORTC-QLQ-C30** • **^I^EORTC-HN-35**	• mouth opening • wight • **^I^oral disorders** • CTCAE mucositis and oral ulcerations • gastric feeding tube • OPSE • pharyngeal phase impairments • PAS • FOIS • pain • EORTC-QLQ-C30 • EORTC-HN-35	• mouth opening • wight • oromotor assessment • CTCAE mucositis and oral ulceration • gastric feeding tube • OPSE • pharyngeal phase impairments • PAS • FOIS • pain • EORTC-QLQ-C30 • EORTC-HN-35
**Mortensen et al. (** 22) **DK** **n=44**	39-77 (58) Pharynx, Larynx, Oral cavity, CUP UICC I-IV Primary Radiotherapy	I: Swallowing exercises at home from RT till 11 month post RT (3x/day, 7 exercises á 10 repetitions), 9 occuptional therapy, exercise diary C: usual care	Not evaluated	• mouth opening • wight • gastric feeding tube • penetration • aspiration • DAHANCA • SPSS • **^c^EORTC-QLQ-C30** • EORTC-HN-35	• mouth opening • wight • gastric feeding tube • penetration • aspiration • DAHANCA • SPSS • **^c^EORTC-QLQ-C30** • EORTC-HN-35	• mouth opening • wight • gastric feeding tube • penetration • aspiration • DAHANCA • SPSS • **^c^EORTC-QLQ-C30** • **^c^EORTC-HN-35**
**Kotz et al. (** 20) **USA** **n=26**	59 all Head and Neck cancers UICC IV Chemoradiotherapy	I: before and during CRT, 1 per week swallowing therapy, 5 swallowing exercises (effortful swallowing, super-supraglottic swallowing, 2 tongue base retraction exercises, Mendelssohn-Maneuver) K: swallowing therapy if necessary	• FOIS • PSS-H&N	• **^I^FOIS** • **^I^PSS-H&N**	• **^I^FOIS** • **^I^PSS-H&N**	• FOIS • PSS-H&N
**Carnaby-Mann et al.** (19) **USA** **n=58**	54+-11.3 Oropharynx T-Stage 1-4 Chemoradiotherapy; Radiotherapy	I: active swallowing exercises (2x/day swallowing therapy, exercises (Falsetto, tongue press exercises, effortful swallowing, TheraBite) and diet C1: usual care C2: 2x/Tag swallowing therapy,,Valchuff”-Maneuver and diet	• **^I^muscle size/composition in MRI and T_2_ -relaxation time** • **^I^mouth opening** • **^I^salivation** • **^I^taste and smell** • wight • Videofluroscopy/aspiration • **^I^swallowing function (MASA)** • **^I^oral feeding** • FOIS • dysphagia related complications	• not evaluated	• muscle size/composition in MRI and T_2_-relaxation time • mouth opening • salivation • taste and smell • wight • Videofluroscopy/aspiration • swallowing function (MASA) • oral feeding • FOIS • dysphagia related complications	• not evaluated

Bold and preceding I: significance in favor of the intervention group.

Bold and preceding C: significance in favor of the control group.

grey: no significant differences.

C (Control group).

CRT (Chemoradiotherapy).

CTCAE (Common Terminology Criteria of Adverse Events).

CUP (Cancer unknown primary).

DAHANCA dysphagia score (Danish Group Head and Neck Cancer).

EORTC-HN35 (European Organization for Research and Treatment of Quality of Life Questionnaire Head and Neck (H&N) -35).

EORTC_QOL_C30 (European Organization for Research and Treatment of Cancer Quality of Life questionnaire C30).

FOIS (Functional Oral Intake Scale).

HADS (Hospital Anxiety and Depression Scale).

H&N (Head and Neck).

I (Intervention group).

MASA (Mann Assessment of Swallowing Abilities).

MDADI (MD Anderson Dysphagia Inventory).

OPSE: Oral Pharyngeal Swallow Efficiency.

PAS (Penetration-Aspiration Scale of Rosenbek).

PSS-HN (Performance Status Scale for Head and Neck Cancer).

RT (Radiotherapy).

SCL-90 (Symptomchecklist-90).

SPSS (Swallowing Performance Status Scale).

Yale Scale (Yale pharyngeal residual severity rating scale).

**Table 4:** Applied exercises in the intervention groups (18–22).

Study	Hajdú (18)	Messing (21)	Mortensen (22)	Kotz (20)	Carnaby-Mann (19)
exercices in Intervention group					
**tongue strength and stretch exercises**	**+**	**+**	**+**		**+**
**lip motor exercises**		**+**			
**chewing**	**+**	**+**	**+**		
**gurgling**	**+**		**+**		
**yawning**	**+**				
**mouth opening**	**+**	**+**			
**Valsalva-Maneuver**	**+**				
**Shaker**	**+**		**+**		
**Mendelsohn-Maneuver**	**+**	**+**		**+**	
**Masako-Maneuver**	**+**	**+**	**+**	**+**	
**effortful swallowing**	**+**	**+**		**+**	**+**
**neck stretching**		**+**			
**TheraBite-System**		**+**			**+**
**Falsetto**			**+**		**+**
**Larynx *range of motion (hold your breath)***			**+**		
**super supraglottic swallowing**				**+**	
**tongue base retraction**	**+**	**+**		**+**	

The RCTs were evaluated using the PEDro-Scale (26, 27). The PEDro scale (27) allows studies to be assessed in terms of their external validity (criterion 1), internal validity (criteria 2 to 9), and the presence of sufficient statistical information to make results interpretable (criterion 10 to 11). It provides a valid measurement tool for assessing the methodological quality of RCTs (23). Accordingly 6 points and more indicate a moderate to high study quality (23, 24).

The risk of bias was evaluated using the Revised Cochrane risk-of-Bias tool for randomized trials (RoB 2) (25). We decided to assess the effect of adhering to intervention in Domain 2 (25)., while using the main outcome parameter of each study.

## 3 Results

**Figure 1:**
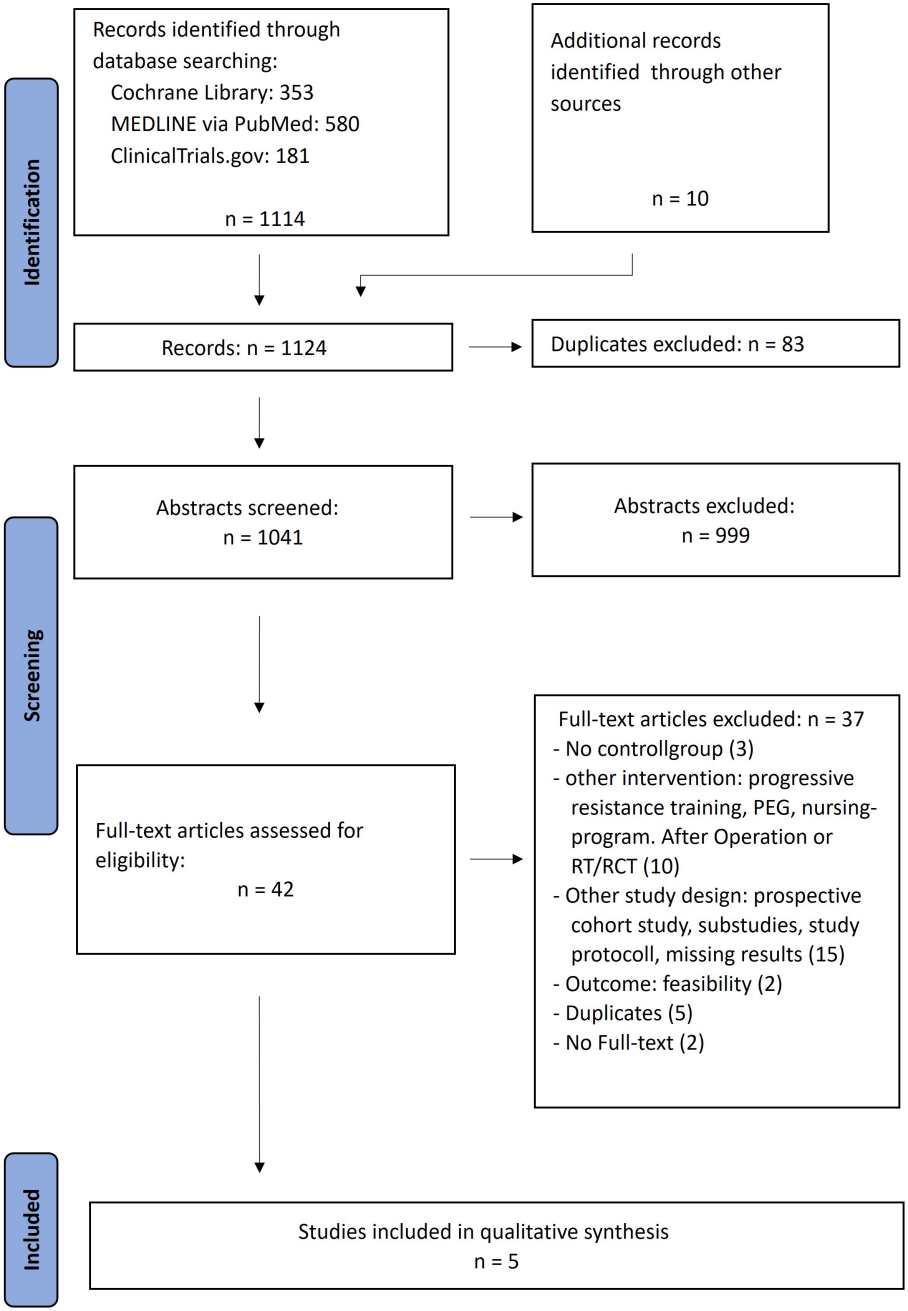
Modified PRISMA flow chart for the representation of the systematic research (own representation, modified according to PRISMA (15)).

1114 studies were identified in the first literature research. 42 studies underwent the full text analysis, from which 5 RCT’s (18–22) finally were evaluated (**Figure 1**).

Only randomized controlled trials that met the PICO criteria were included in the further analysis. In view of the specific research question regarding the efficacy of preventive exercise therapy measures, studies investigating enteral versus oral nutrition during radiotherapy or adherence or feasibility as an outcome were excluded from the 41 hits. Similarly, sub-studies and studies without available results were excluded. These included study protocols, reports of preliminary data, or the follow-up study by Kraaijenga et al. (28), which no longer differentiated between the intervention and control groups of the underlying study by Kotz et al. (20). When updates to studies were available, the current results were chosen for further evaluation. Van der Molen et al. (29, 30) investigated the effectiveness of a prevention program using the TheraBite^®^ Jaw Motion Rehabilitation System™ compared to standard care. Because the study was conducted in the Netherlands, where prehabilitation therapy is already part of usual care (30), it did not meet the PICO criterion of no preventive exercises as a comparison, so this study was excluded. The studies by Virani et al. and Wall et al. (31) also lacked comparison groups without preventive interventions. Three other studies did not meet the inclusion criterion of starting prehospital interventions because they were postoperative or after radiotherapy (32–34). Only preliminary data and study protocol are available for the Redyor randomized controlled trial (35), which collected data in 2018-2019 to review preventive swallowing exercises (35–37). Because full text has not yet been published on the study results, the study was excluded.

It should be mentioned that criteria five and six of the PEDro-scale cannot be matched as blinding is not possible, neither for participants nor therapists, due to the nature of the evaluated treatment. However, the studies of Hajdú et al. (18), Messing et al. (21), Kotz et al. (20) and Carnaby-Mann et al. (19) achieve 6 or 7 points, i.e. showing a moderate to high quality (see **Table 5**). Only the study of Mortensen et al. (22) achieves 4 points and therefore reveals less quality and validity.

**Table 5:** Quality of the selected studies according to the PEDro scale.

PEDro-criteria	1	2	3	4	5	6	7	8	9	10	11	Total score
**Studies**												
**Hajdú et al.** (18)	**+**	**+**	**+**	**+**	**-**	**-**	**-**	**-**	**+**	**+**	**+**	**6**
**Messing et al.** (21)	**+**	**+**	**+**	**+**	**-**	**-**	**-**	**-**	**+**	**+**	**+**	**6**
**Mortensen et al.** (22)	**+**	**+**	**-**	**+**	**-**	**-**	**-**	**-**	**-**	**+**	**+**	**4**
**Kotz et al.** (20)	**+**	**+**	**-**	**+**	**-**	**-**	**-**	**+**	**+**	**+**	**+**	**6**
**Carnaby-Mann et al.** (19)	**+**	**+**	**+**	**+**	**-**	**-**	**+**	**-**	**+**	**+**	**+**	**7**

Details of the included RCTs are presented in **Table 3**. Tumor stages and localizations are distributed heterogeneously, same is true for the intervention and outcome parameters. The common denominator of the evaluated RCTs is the treatment of primary radiation or chemoradiation. Collectives that underwent primary surgery were not yet investigated. Outcome measurement tools include physiological parameters, such as muscle thickness/muscle size and its composition in magnetic resonance imaging (19), oral motor function (21), mouth opening (18, 19, 21, 22), swallowing function parameters collected by FEES (Flexible Endoscopic Evaluation of Swallowing) (18, 19) or VFSS (Video fluoroscopic Swallowing Study) (19, 21), for example, using the PAS (Penetration-Aspiration Scale of Rosenbek) (18, 19, 21) and Yale pharyngeal residual severity rating scale (18), feeding-related parameters, such as tube dependence, weight (22), and dietary form (19), collected with FOIS (Functional Oral Intake Scale) (18, 20, 21), SPSS (Swallowing Performance Status Scale) (22) and MASA (Mann Assessment of Swallowing Abilities) (19).

Questionnaires were used to assess general (EORTC_QOL_C30/HN35) (13, 18, 21, 22) and swallowing-related quality of life (MDADI) (18), depression and anxiety (HADS, SCL-95) (13, 18). The occurrence of complications such as pneumonia or dehydration was also assessed (19).

The interventions used in the RCTs (18–22) demonstrate a strong heterogeneity (**Table 4**). Tongue motor and strengthening exercises, the Masako maneuver, and forceful swallowing were most frequently used as preventive exercises. The selected exercise frequencies and repetition rates are not justified in the studies (18, 19, 21, 22). Only Kotz et al. (20) critically comment that there is no evidence for the appropriate dose of swallowing exercises and that the performance of three sets of ten repetitions of each exercise was arbitrarily set. They note that performing the exercises three times daily could be associated with “breakfast, lunch and dinner or morning, noon and night” to support compliance (20).

Significant group differences in favor of the intervention group were found at different measurement time points. Hajdú et al. (mouth opening, anxiety, pain and QoL) (18) and Carnaby-Mann et al. (muscle composition an T2 relaxion time, swallowing function, oral feeding, mouth opening, salivation, sense of taste and smell) (19) after 6 weeks. After 2 to 3 months in QoL (18, 21), oral feeding (18, 20, 21) and after 6 months in oral motor function (21) and oral feeding (20). Only Messing at al. show a significant better mouth opening 24 months after therapy (21), there were no differences between groups in the long term follow up in the other studies (18–20, 22). Mortensen et al. show significant better outcome in parts of QoL in the control group (22).

All studies indicate that adherence to exercise treatment in the intervention groups decreases over time; drop-out rates range from 25% (18) to 49% (22) within the study period. Among the reasons for discontinuing exercise, severe therapy-associated pain in the mouth, throat discomfort, and general fatigue were mentioned (20). Mortensen et al. refer to the publication by Shinn et al. (38) and describe “lack of understanding of the importance of swallowing exercises, the effort involved, pain, and forgetfulness” as causes of poor adherence (22).

The overall risk of bias is high in all studies (see **Table 6**). They all have a low risk of bias in the randomization process (Domain 1) and the reported result (Domain 5). Three studies (18, 21, 22) present some concerns and two (19, 20) high risk of bias in Domain 2, where we decided to assess the effect of adhering to intervention. Because of high dropout rates (18, 19, 21, 22) there is a high risk of bias in Domain 3 (missing outcome data). When the outcome is patient reported (20, 21), then there are some concerns in Domain 4 (risk of bias in measurement of the outcome).

**Table 6:** Risk of bias assessment using Revised Cochrane risk-of-bias tool for randomized trials (RoB 2) (25).

Study	Domain1	Domain2	Domain3	Domain4	Domain5	Overall
**Mortensen et al.** (22)	Low	Some concerns	High	Some concerns	Low	High
**Messing et al.** (21)	Low	Some concerns	High	Low	Low	High
**Hajdú et al.** (18)	Low	Some concerns	High	Low	low	High
**Carnaby-Mann et al.** (19)	Low	High	High	Low	Low	High
**Kotz et al.** (20)	Low	High	Low	Some concerns	Low	High

Domain 1: Risk of bias arising from the randomization process.

Domain 2: Risk of bias due to deviations from the intended interventions (effect of adhering to intervention).

Domain 3: Missing outcome data.

Domain 4: Risk of bias in measurement of the outcome.

Domain 5: Risk of bias in selection of the reported result.

Overall risk of bias.

## 4 Discussion

Despite all efforts for a rapid diagnosis and initiation of therapy in cases of suspected HNC, there are unused time windows in the diagnostic phase, namely the waiting period until the upper airway and esophagus can be examined under general anesthesia (panendoscopy) and the subsequent phase of therapy planning. Thus, on average, there is a period of two to four weeks that would lend itself to targeted prehabilitation without delaying therapy.

The need for identification of critical and prognostic swallowing disorders may be substantial if more than a half of the patients at a typical head and neck tumor center suffer from dysphagia (12). In subgroups, specifically concerning oropharyngeal carcinomas, such disorders also occur in up to 80% of cases. This effect is particularly relevant because the proportion of younger patients in this group increases due to the association with human papillomavirus (13, 39). Thus, it has already been shown that marked postoperative dysphagia without the ability to take oral food is an early indicator of poorer survival regardless of tumor stage (40). In addition, aspiration pneumonia may have prognostic significance, with a three- to fourfold increased incidence in HNC patients compared with a control group, as shown by data from the American SEER registry (11).

The detection of nutrition-related factors and their management in prehabilitation programs is already considered essential because of their prognostic importance (3). The European Society for Clinical Nutrition and Metabolism mentioned important aspects in guidelines for nutritional management in cancer patients. Before the therapy started all patients should be screened for their risk of malnutrition or for their body mass index, respectively. If necessary, this is followed by a detailed nutritional assessment and multimodal individualized intervention to increase dietary intake and physical activity (41).

In order to compare our results, we searched for other reviews on these topics and found four (13, 14, 42, 43) more review articles that examined not only randomized studies but also non-randomized studies. The heterogeneity in intervention and outcome parameters is also reflected in these papers as well as the high risk of bias (13, 14, 42, 43).

Little attention has been paid to prehabilitation aspects in HNC patients, although they may show organ-specific risk factors of tumor- or therapy-related oropharyngeal dysphagia. Therefore, swallow-specific intervention could be an essential component within a multimodal prehabilitation approach. The present systematic evidence review shows initial success in this area, but also several limitations. There is consensus that dysphagia should be treated as early as possible, even if an “early” start of intervention is interpreted variably in the studies reviewed (13, 42).

In comparison to the control group there were short term effects in the prehabilitation-groups, such as better QoL 2 or 3 months after therapy (18, 21) and better mouth opening after therapy (18, 19), but no long-term effects were found. Interestingly there are also conflicting results in QoL reported in some studies (21, 22).

Several factors could have a moderating influence on the effectiveness of the intervention, one being whether the therapy is delivered in person or in the form of written exercise instructions (20). Studies evaluating the relationships of delivery mode, patient-related factors, and therapy adherence in HNC patients show that professionally guided therapies achieve the best adherence in the first three weeks, while an app-assisted version still leads to better adherence than letting the patient practice alone. Nicotine use at intervention onset and concurrent chemotherapy in the setting of primary radiotherapy were found to be significant negative predictors of adherence (31). Moreover, clinically relevant anxiety or depression symptoms are regularly associated with dysphagia, in almost 50% of cases (44), unsurprisingly given the central social importance of eating and drinking together. This important influence as well as outcome parameter should be considered in the design of future studies.

All efforts at preventive measures must take the deficit in health literacy into account, especially among HNC patients (45). It remains essential to inform patients before tumor treatment of possible consequences, such as dysphagia, and of ways to show them self-efficacious methods to maintain their health and prevent further symptoms (46).

### 4.1 Limitations of evidence

After all, several studies of moderate to high quality are available, even if we see a high risk of bias in the individual studies, caused by the lack of opportunity for blinding due to the intervention and the lack of adherence of the study participants. Not only the rather small study populations and high dropout rates limit the validity of the studies, but also the existing large heterogeneity regarding the interventions and outcome parameters impede a metaanalysis (14, 43). Evidence is further limited by the large differences in inclusion and exclusion criteria and measurement time points, which make a reliable assessment difficult. Thus, a clear statement is neither possible regarding the efficacy of preventive measures nor concerning the optimal intervention timing, intervention duration and frequency, as well as exercise selection (13). A similar issue exists in neurological swallowing rehabilitation, where evidence for the correct or most effective number and frequency of swallowing exercises is also lacking (47).

The majority of publications only account for patients that were treated with radiation and chemoradiation treatment, surgically treated patients were not considered. In Germany, surgery often precedes adjuvant radio- or chemo-radiotherapy in an early or selected high tumor stage, whereas primary radio- or chemo-radiotherapy is frequently implemented in advanced tumor stages primarily (45, 48). Study results from collectives, that were exclusively irradiated, must not be transferred to representative German collectives of patients, because QoL and swallowing function are heavily influenced by the chosen treatment (12).

The research project titled “The Effects of Phoniatric Prehabilitation in Head and Neck Cancer Patients on Aspiration and Preservation of Swallowing (PREHAPS)” (DRKS00029676), sponsored by G-BA (Gemeinsamer Bundesausschuss) is partly based on this systematic review. PREHAPS provides a prospective randomized trial that investigates the prehabilitation of swallowing disorders of patients at a German Head-Neck-cancer-center for the first time.

In order to utilize the potential advantages of prehabilitation according to the needs of HNC patients, additional human resources (especially speech therapy, phoniatrics) have to be provided, which are currently not refinanced in the German health care system. However, studies indicate that care costs even can be reduced (49, 50) and that early rehabilitation of swallowing disorders can mitigate the financial consequences of the disease (51). In selected populations, the combination of prehabilitation and early rehabilitation has been shown to be less costly than the traditional symptom-only approach (52).

### 4.2 Limitations of the review process

The review process was first carried out by only one person (second author A.M., professional speech language therapist) in the sense of an exploratory literature search, which is a limitation of the methodology presented here. All included articles were read by all authors and discussed in the working group.

## 5 Conclusion

Prehabilitation is becoming increasingly important in oncology, and the prognostic significance of dysphagia has been recognized, particularly in the treatment of head and neck tumors. However, the efficacy of prehabilitative interventions has been only rudimentarily investigated. Active exercises of swallowing function may lead to demonstrably better outcomes immediately after radio(-chemo)-therapy, although evidence of long-term benefit is lacking to date. Preventive exercises provide the possibility of reducing the consequences of dysphagia, maintaining swallowing function, and improving quality of life. All currently available studies exclusively investigated patients with primary radiotherapy. High-quality research that also focuses on patient collectives including surgical treatment strategies are therefore urgently needed. It is of great importance to investigate questions of a suitable prehabilitation approach in particular, regarding the selection of patients, the start of therapy, the form of therapy, and the selection and frequency of exercise.

## Author contributions

SV: Visualization, Writing – original draft. AM: Data curation, Formal analysis, Visualization, Writing – original draft. JM: Resources, Writing – review & editing. CS: Resources, Writing – review & editing. PK: Methodology, Supervision, Validation, Writing – review & editing. JK: Project administration, Supervision, Writing – review & editing.
